# Allelopathic Activity of the Invasive Plant *Polygonum chinense* Linn. and Its Allelopathic Substances

**DOI:** 10.3390/plants12162968

**Published:** 2023-08-17

**Authors:** Thang Lam Lun, Shunya Tojo, Toshiaki Teruya, Hisashi Kato-Noguchi

**Affiliations:** 1 Department of Applied Biological Science, Faculty of Agriculture, Kagawa University, Miki 761-0795, Kagawa, Japan; tllunpi43@gmail.com; 2 The United Graduate School of Agricultural Sciences, Ehime University, Matsuyama 790-8566, Ehime, Japan; 3 Graduate School of Engineering and Science, University of the Ryukyus, Nishihara 903-0213, Okinawa, Japan; k218370@eve.u-ryukyu.ac.jp; 4 Faculty of Education, University of the Ryukyus, Nishihara 903-0213, Okinawa, Japan; t-teruya@edu.u-ryukyu.ac.jp

**Keywords:** *Polygonum chinense*, invasive plant, allelopathic substances

## Abstract

*Polygonum chinense* Linn., belonging to the Polygonaceae family, is distributed mostly in northern temperate climates. This species is a high-risk invasive plant and is thought to possess allelopathic potential. This study aimed to isolate and identify the allelopathic substances from *P. chinense*. Aqueous methanol extracts of *P. chinense* significantly inhibited the growth of alfalfa and Italian ryegrass seedlings in a species- and concentration-dependent manner. Activity-guided fractionation led to the isolation of two active compounds: dehydrovomifoliol and loliolide. A cress bioassay was used to determine the biological activity of dehydrovomifoliol, and cress, alfalfa, and Italian ryegrass were used to determine loliolide. Dehydrovomifoliol significantly suppressed the seedling growth of cress at the concentration of 1 mM, and the concentrations necessary for 50% growth inhibition (I_50_ values) of the roots and shoots were 1.2 and 2 mM, respectively. Loliolide significantly suppressed the shoot growth of cress, alfalfa, and Italian ryegrass at the concentration of 1 mM, and the concentrations necessary for I_50_ values of the shoots and roots were 0.15 to 2.33 and 0.33 to 2.23 mM, respectively. The findings of our study suggest the extracts of *P. chinense* might have growth-inhibitory potential and that dehydrovomifoliol and loliolide might contribute as allelopathic agents.

## 1. Introduction

**Figure 1:**
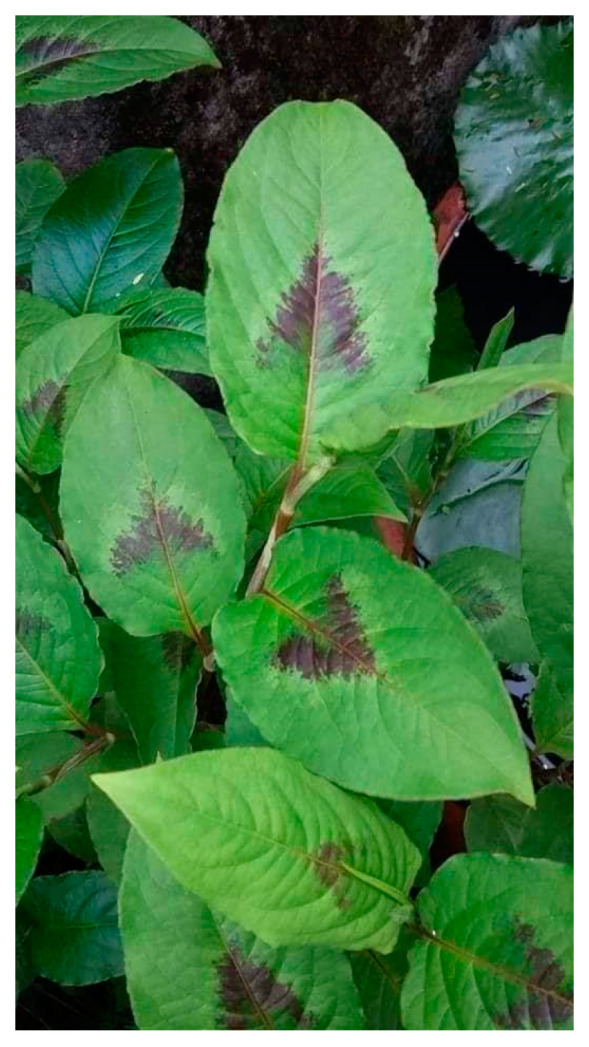
*Polygonum chinense*.

*Polygonum chinense* Linn., a rhizomatous herbaceous perennial from the Polygonaceae family, is commonly known as Chinese knotweed or smartweed (Figure 1). *Polygonum chinense* is widespread across Vietnam, Bhutan, Taiwan, China, Indian Subcontinent, Japan, South Korea, North Korea, Indonesia, Malaysia, Nepal, Papua New Guinea, the Philippines, Sri Lanka, Myanmar (Burma), and Thailand [1]. *Polygonum chinense* can be found in disturbed areas such as home gardens, abandoned gardens, and roadsides [1,2]. In natural environments, it can be found in forests and on riverbanks, and grows from sea level to 3000 m [3], especially in areas of regrowth and natural clearings [1,4,5], where it may disrupt forest regeneration [5]. This species is used in herbal remedies, such as for the treatment of sore throat, dysentery, and enteritis in Malaysia and Vietnam [6], and for the treatment of skin diseases and inflammation in traditional medicine in Myanmar [7], and in India, Japan, China, and Southeast Asian countries [8,9,10]. On the other hand, this species is a high-risk invasive species and tolerates a wide range of environmental conditions in Asia and coastal areas of New South Wales and Queensland in Australia [11]. The *P. chinense* grows up to approximately 1 to 1.5 m and has prominent nodes. Their stems and branches are spineless, reddish-purple, and lack hair arranged with alternate leaves and ovate to oblong shape. The size of the flowers is small, and their colors are white or light reddish and arranged in capitate, inflorescence axillary. Fruits are berries, shaped of globose with black and small seeds. The plant grows rapidly, forming thick canopies that can smother native plants [2], and is perceived as an environmental weed that suppresses other plant species with its dense mats [5,12,13].

*Polygonum chinense* is one of the undesirable weeds that continually evolve, survive, thrive, and reproduce weeds in tea plantations and can infest severely in mature tea [14,15], especially in southern India [16] where it blocks the drainage systems and covers the tea bushes. It could have the potential to affect nursery operations, orchards, and forestry [17], and it is listed as an “agricultural weed” in Thailand and Taiwan [12]. Allelochemicals produced by invasive weed species disrupt the fundamental physiological processes of crops, restricting their growth and development. Consequently, weeds compete with crops for environmental resources [18]. Compared with other plant species, weeds have more genetic variety and phenotypic plasticity [19,20,21].

Traditional explanations for biological invasions rely on several premises, including enemy release hypotheses, disturbance, biotic resistance, and so on [22]. Invasive plants may have particularly potent allelopathic effects, according to the novel weapons hypothesis [21,23], because introduced plants did not coevolve with the invader and are therefore more vulnerable to its allelopathic compounds than native plants [20]. Allelopathic effects play a role in the success of various plant invaders, such as spotted knapweed [24,25], garlic mustard [26,27], Canada golden rod [28,29], sticky snakeroot [30], bitter bush [31], and Japanese knotweed [23,32]. By releasing chemical compounds into the environment, such invasive exotic species displace local plants, making it easier for the invasive species to become established [24,33,34]. In growth assays, aqueous extracts of knotweed species inhibit other plant species [35,36], which suggested that invasive knotweeds may produce allelochemicals that act as novel weapons and facilitate monodominance. *Polygonum chinense* is of particular interest experimentally because it has the ability not only to invade forests, habitats that are typically considered to be relatively suppressed by other plant species with their dense mats, but also to be used as herbal remedies. In our previous study, aqueous methanol extracts of *P. chinense* showed growth-inhibitory activity, and two inhibitory compounds were isolated [37]. Notably, there were other active fractions that may contain other inhibitory substances. Accordingly, this experiment was conducted to evaluate the growth-inhibitory activity of *P. chinense* extracts on another two test plants, to isolate the plant growth-inhibitory substances, and to assess the activity of the growth-inhibitory substances.

## 2. Results

### 2.1. Allelopathic Activity of the Polygonum chinense Plants

**Figure 2:**
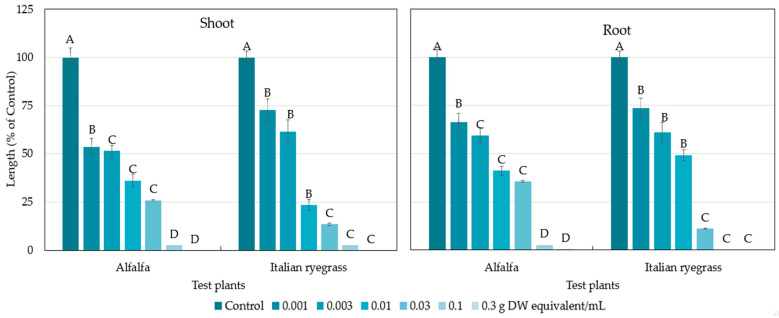
Effect of the *Polygonum chinense* above plant parts extracts on the seedling growth of alfalfa and Italian ryegrass at six concentrations. Each bar expresses mean ± SE with six replicates (*n* = 60). The letters on the bars signify significant differences (Tukey’s HSD test, at 0.05 probability level).

The extracts of P. chinense above plant parts inhibited the shoot and root growth of both the test plants (alfalfa and Italian ryegrass) at the lowest concentration of 0.001 g dry weight (DW) equivalent extract/mL (Figure 2). The concentration of 0.01 g DW equivalent extract/mL of P. chinense suppressed the shoot growth of alfalfa and Italian ryegrass to 36.18 and 23.47% of the control, respectively, and suppressed the root growth to 41.13 and 49.19%, respectively. The extract obtained from 0.3 g of P. chinense completely suppressed the shoot and root growth of alfalfa and Italian ryegrass. The concentrations needed to inhibit the growth of the alfalfa and Italian ryegrass shoots by 50% (I_50_ values) were 0.0035 and 0.043 g DW equivalent extract/mL, respectively, and 0.0049 and 0.0052 for the root growth (Table 1), respectively.

### 2.2. Isolation and Purification of the Allelopathic Substances

**Figure 3:**
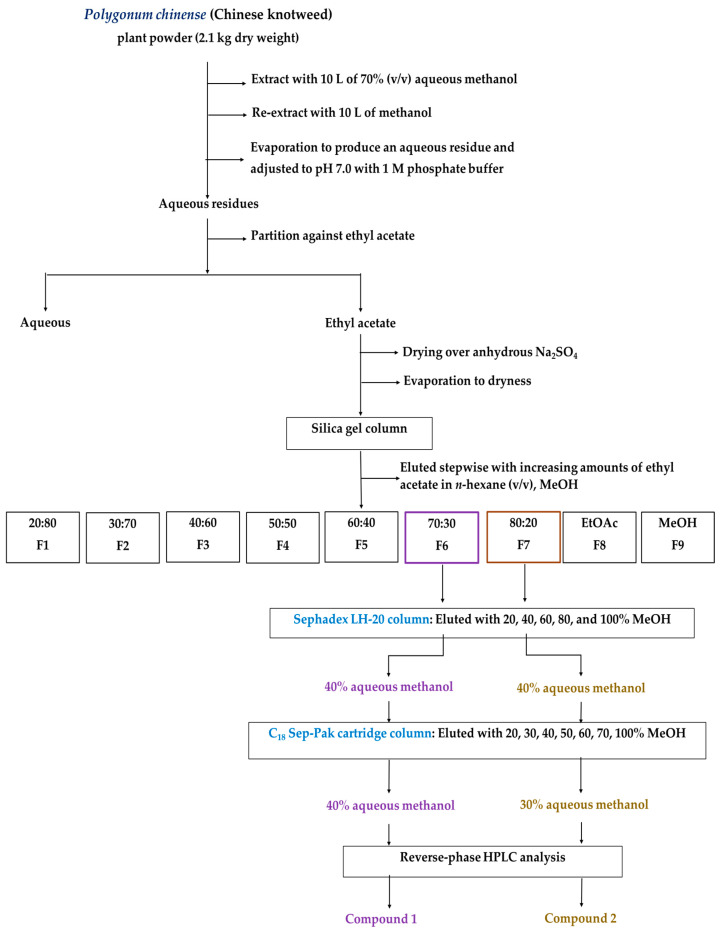
Procedure for isolation and purification of allelopathic active compounds from the extracts of the above plant parts *Polygonum chinense*.

**Figure 4:**
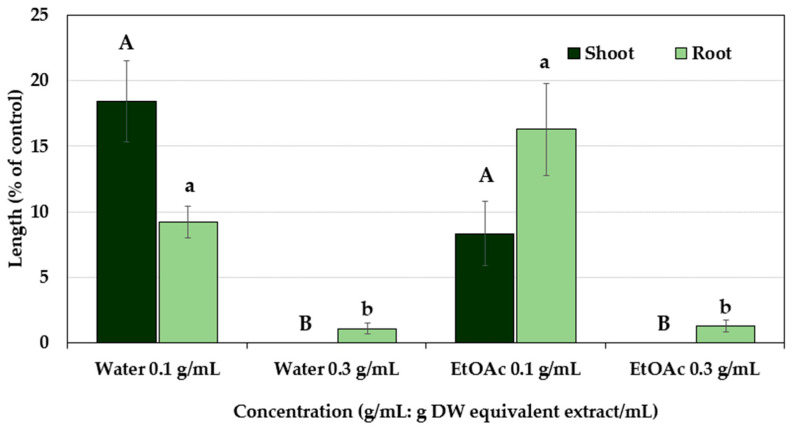
Effects of the distilled water (aqueous) and ethyl acetate (EtOAc) fractions on the seedling growth of cress obtained by partitioning the extracts of *Polygonum chinense* at the concentrations of 0.1 and 0.3 g DW equivalent extract/mL. Each bar expresses mean ± standard error with three replicates (*n* = 30). Different letters on the bars signify significant differences (Tukey’s HSD test, at 0.05 probability level).

**Figure 5:**
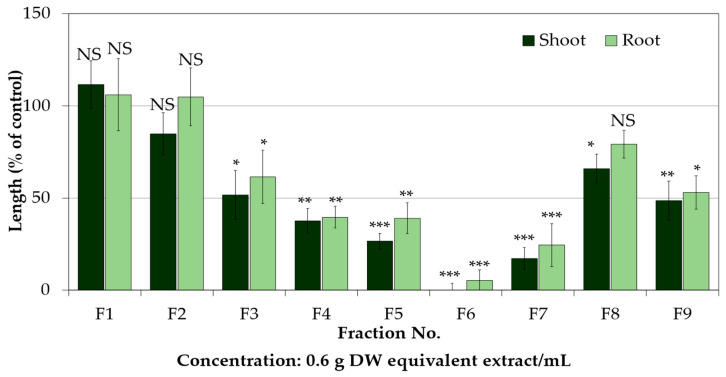
The inhibitory effect of the *P. chinense* above plant parts extracts on the shoot and root growth of cress. The cress seeds were treated with different ratios of EtOAc in n-hexane (*v*/*v*) at the concentration of 0.6 g DW equivalent extract/mL using nine fractions and the last fraction eluted with MeOH. Each bar expresses mean ± standard error with three replicates (*n* = 30). *, **, *** Asterisks signify significant differences (Tukey’s HSD test at 0.05, 0.01, 0.001 probability level, respectively). NS: Non-significant differences.

A schematic diagram of the isolation procedure of the substances is shown in Figure 3. The ethyl acetate and aqueous fractions of the P. chinense extracts retarded the seedling growth of the cress in a dose-dependent manner (Figure 4). At the concentration of 0.3 g DW equivalent extract/mL, both fractions completely inhibited the shoot growth of the cress and inhibited the root growth to less than 2% of the control. The ethyl acetate fraction was subjected to further purification steps using a column of silica gel, resulting in fraction 6 (F6) showing the most inhibitory activity followed by fractions F7, F5, F4, F9, and so on (Figure 5). Therefore, the most active fraction of F6 and the second active fraction of F7 were chosen for continuing the purification process and purified through a Sephadex LH-20 column and reverse-phase C_18_ cartridges, and finally, two active compounds were isolated by using reverse-phase HPLC, which was characterized by analyzing the spectral data.

### 2.3. Characterization and Biological Activity of Compound ***1***

**Figure 6:**
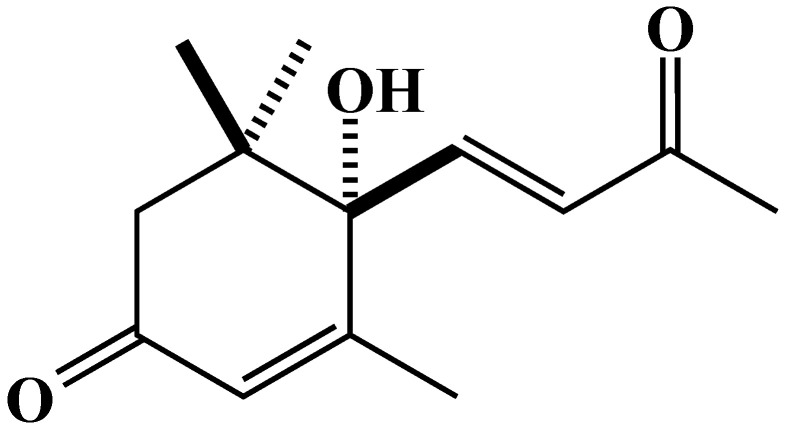
The structure of dehydrovomifoliol isolated from the above plant parts of *P. chinense* extract.

The molecular formula of compound **1**, a colorless oil, was found to be C_13_H_17_O_3_ as determined by HRESIMS (high-resolution electrospray ionization mass spectroscopy) *m*/*z* 245.1147 [M + Na]^+^ (calcd for C_13_H_18_O_3_Na 245.1148). The ^1^H NMR (proton nuclear magnetic resonance) spectrum of compound **1** as measured in CD_3_OD showed four methyl proton signals at *δ*_H_ 2.31 (3H, s), 1.90 (3H, s), 1.06 (3H, s), and 1.02 (3H, s); three olefinic proton signals at *δ*_H_ 6.99 (1H, d, *J* = 15.8), 6.44 (1H, d, *J* = 15.8), and 5.94 (1H, s); and two methylene proton signals at *δ*_H_ 2.60 (1H, d, *J* = 17.2) and 2.28 (1H, d, *J* = 17.2). The ^1^H NMR spectrum of compound **1** was in agreement with the reported data of dehydrovomifoliol (Figure 6) [38].

**Figure 7:**
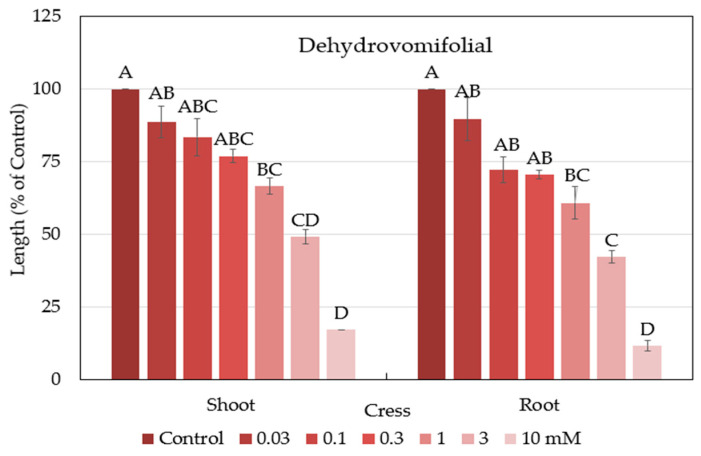
Effects of dehydrovomifoliol against the growth of cress seedlings. Each bar expresses mean ± SE with three replicates *(n* = 30). Different letters on the bars signify significant differences, but any two means having a common letter are not significant differences within the group (Turkey’s HSD test, at 0.05 probability level).

The biological activity of dehydrovomifoliol against cress was assayed. The results of the assay showed that the seedling growth of cress was significantly retarded at the concentration of 1 mM (Figure 7). At the concentration of 3 mM, dehydrovomifoliol suppressed the growth of seedlings by more than 50% of the control whilst at the concentration of 10 mM by more than 75% of the control. The I_50_ values for dehydrovomifoliol against the cress seedling roots and shoots were 1.2 and 2 mM (Table 2), respectively.

### 2.4. Characterization and Biological Activity of Compound ***2***

**Figure 8:**
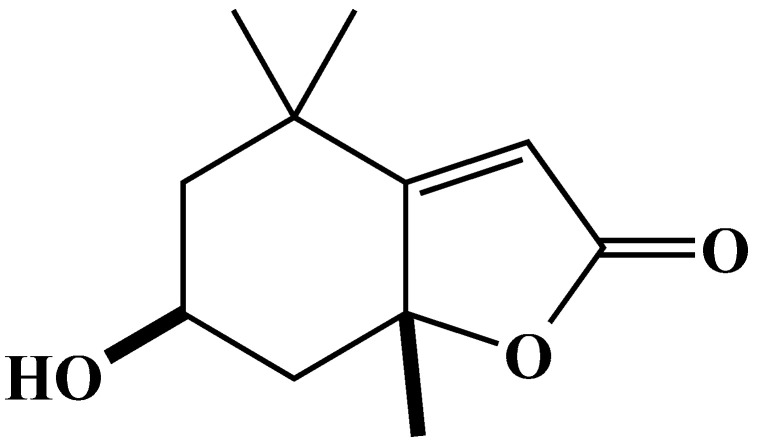
The structure of loliolide isolated from the above plant parts of *P. chinense* extract.

Compound **2** was an amorphous powder; [α]_D_^24^ = −82.8 (*c* 0.54, MeOH). Its molecular formula was found to be C_11_H_16_O_3_ as determined by HRESIMS *m*/*z* 197.1171 [M + H]^+^ (calcd for C_11_H_17_O_3_, 197.1172). The ^1^H NMR spectrum of compound **2** as measured in CD_3_OD showed three methyl proton signals at *δ*_H_ 1.76 (3H, s), 1.47 (3H, s), and 1.28 (3H, s); one olefinic proton signal at *δ*_H_ 5.75 (1H, s); one methine proton signal at *δ*_H_ 4.22 (1H, m); and four methylene proton signals at *δ*_H_ 2.42 (1H, dt, *J* = 13.8, 2.7), 1.99 (1H, dt, *J* = 14.4, 2.6), 1.75 (1H, dd, *J* = 13.8, 4.0), and 1.53 (1H, dd, *J* = 14.4, 3.7). The ^1^H NMR spectrum of compound **2** was in agreement with the reported data of loliolide (Figure 8) [39].

**Figure 9:**
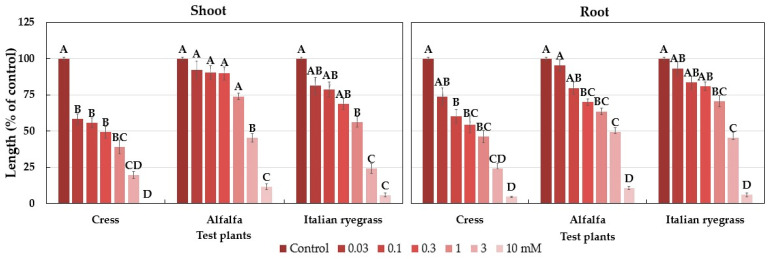
Effects of loliolide against the growth of cress, alfalfa, and Italian ryegrass. Each bar expresses mean ± standard error with three replicates (*n* = 30). Different letters on the bars signify significant differences, and any two means having a common letter are not significant differences within the group (Turkey’s HSD test, at 0.05 probability level).

The biological activity of loliolide was evaluated against cress, alfalfa, and Italian ryegrass. The results from the bioassays showed that the cress seedlings were significantly suppressed at the concentration of 0.03 mM, and the alfalfa and Italian ryegrass seedlings were significantly suppressed at the concentration of 1 mM (Figure 9). The concentration of 10 mM completely suppressed the shoot growth of cress and that of the alfalfa and Italian ryegrass plants to less than 15% of control, whilst the root growth of the three test plants was suppressed to less than 15% of control. The I_50_ values for loliolide against the shoots of the test seedlings were in the range of 0.15 to 2.33 mM and for the roots were in the range of 0.33 to 2.23 mM (Table 3).

## 3. Discussion

The above plant parts extracts of *Polygonum chinense* (Chinese knotweed) significantly inhibited the growth of alfalfa and Italian ryegrass (Figure 2). Our previous experiment revealed that *P. chinense* plant extracts inhibited four test plants (cress, lettuce, timothy, and barnyard grass) [37]. The inhibitory effects of the plant extracts against the test plants increased with increasing extract concentration. Allelopathic substances, which are released by alien invasive plants, affect the germination of seeds and the development of native species [40,41]. The I_50_ values show that the inhibition by the plant extracts differed depending on the test plant species (Table 1). Such species-specific and dose-dependent inhibition has also been documented in other studies [42,43,44]. The findings are consistent with the earlier studies reporting that the inhibitory effect depended on the concentration of extracts, and the sensitivity to the extracts relied on the biochemical and physiological characteristics of each plant species [45,46]. Differences in the biochemical and physiological nature of test plants may be responsible for the inhibitory effects of the extracts [47]. Isolating and identifying secondary metabolites from natural sources (plants) is crucial for the development of ecologically friendly natural herbicides. In our previous experiment, two active compounds, (-)-3-hydroxy-*β*-ionone and (-)-3-hydroxy-7,8-dihydro-*β*-ionone, were isolated and identified from *P. chinense* plant extracts, and both identified compounds significantly retarded the seedling growth of cress [37]. In the present study on the *P. chinense* above plant parts extracts, two other active compounds were purified and characterized as dehydrovomifoliol (Figure 6) and loliolide (Figure 8) using several chromatographic methods (Figure 3).

Both compounds are reported as nor isoprenoids, and their antimicrobial, antiproliferative, anti-algal, antioxidant, and cytotoxic properties have been explored [48,49]. There are several different plant species that contain dehydrovomifoliol: *Phaseolus vulgaris* L. [50], *Helianthus annuus* L. cv. Peredovick [51], *Beta vulgaris* var. cicla [52], *Malva silvestris* [53], *Cucumis sativa* [38], *Arctium lappa* L. [54], *Raphanus sativus* L. [55], and *Dregea volubilis* (L.f.) Benth. ex. Hook. f. [43], and it has also been synthesized from C_9_-hydroxy ketone [56]. Ren et al. [57] reported that dehydrovomifoliol has cytotoxic effects against human cancer cells. Hodges and Porte (1964) reported firstly the loliolide compound from *Lolium perenne* L. and its diverse biological activities [58,59,60]. Since then, it has been found in different plant and animal species, in both land and sea ecosystems [61], such as *Bunias orientalis* L. [62], *Centrostachys aquatica* (R.Br.) Moq. [63], *Digitaria sanguinalis* (L.) Scop. [64], *Marsilea crenata* C. Presl [65], and *Albizia richardiana* (Voigt.) King and Prain [66], and it has also been obtained by synthesizing C_11_-aldehyde [56]. However, the isolation of dehydrovomifoliol and loliolide from *Polygonum chinense* has not yet been documented in the literature.

The results of our study showed that dehydrovomifoliol inhibited the growth of cress (Figure 7), and loliolide inhibited the growth of cress, alfalfa, and Italian ryegrass (Figure 9). The level of inhibition differed depending on the extract concentration, the species of the test plants, and the chemical structures of the compounds. Differences in allelopathic activity may be led by variations in the chemical structures of substances [67]. Numerous invading plant species have been found to be allelopathic, and their phytotoxic compounds have adverse effects on other plant species [68,69,70]. According to Dayan et al. [71], the varying levels of bio-effectiveness among the compounds may be due to the distinct mode of action that different compound structures have on target plants. The I_50_ values of loliolide (Table 3) showed the inhibitory effect differed with plant species. Dehydrovomifoliol is a C_13_-nor isoprenoid with two oxo groups at C-3 and C-9 and a hydroxyl group in the benzene ring. Loliolide, on the other hand, is a C_11_-nor isoprenoid structured in a benzene ring with a hydroxyl group at the C-3 position and one oxo group at the C-8 position (Figure 8). The I_50_ values of the two compounds show the cress seedlings were more sensitive to loliolide than dehydrovomifoliol. These results support the hypothesis of Kobayashi et al. [72] that the phytotoxic potential of loliolide may be due to the hydroxyl group at the C-3 position. The chemical structure, including the number and position of different functional groups replaced in the benzene ring, controls the toxicity of phytochemicals [73,74]. The findings in the present study designate that dehydrovomifoliol and loliolide have growth-inhibitory activity and may contribute to the allelopathic effect of *P. chinense*. As a result, *P. chinense* could be used as a soil additive resource or soil enhancement to manage weeds in an environmentally acceptable manner. Its plant residues may also be discharged into the soil and behave as allelopathic chemicals.

## 4. Materials and Methods

### 4.1. Extraction and Plant Material

Samples of *Polygonum chinense* were collected in different areas of Mandalay Region, Myanmar from July to August 2020 (Figure 1). After removing dust and contamination, all the samples were air-dried and ground. Ground dried powder (50 g) of the above plant parts of *P. chinense* was extracted using 300 mL of a mixture of distilled water and methanol (MeOH) in a ratio of 30:70 (*v*/*v*) for a couple of days. The crude extracts were filtrated through No. 2 filter paper (Toyo Roshi Kaisha Ltd., Tokyo, Japan), and the residues were re-extracted using 300 mL of MeOH for a day and filtrated again. Both filtrates were combined in one flask and evaporated until dry in a 40 °C vacuum using a rotary evaporator to produce the concentrated crude extract.

### 4.2. Growth-Inhibitory Assay

*Medicago sativa* L. (alfalfa) and *Lolium multiflorum* Lam. (Italian ryegrass) were selected to determine growth-inhibitory activity. The concentrated extract of *P. chinense* was diluted in 100 mL MeOH. Six different concentrations (0.001, 0.003, 0.01, 0.03, 0.1, and 0.3 g dry weight (DW) equivalent extract/mL) were used to evaluate the growth-inhibitory assay of the extracts on the test plants, and the exact extract amount was put on filter papers in each 2.8 cm Petri dishes. After the extract concentration was dried, the aqueous solution of 0.6 mL of 0.05% (*v*/*v*) of Tween 20 (polyoxyethylene sorbitan monolaurate; Nacalai Tesque, Inc., Kyoto, Japan) was put into the Petri dishes to moisten the filter paper, and then 10 dicot seeds of alfalfa and 10 monocot seeds of sprouted Italian ryegrass were placed in the Petri dishes. Only Tween 20 aqueous solutions were used for the control treatment. After incubation for a couple of days in darkness, seedling length was measured. The shoot and root growth of the test plants were measured by using a ruler. The inhibition was calculated by comparing the treatments of the extracts with the control of each test plant. To compute the inhibition % of seedling growth, the following formula was used.

$$(\%)seedling\;growth=1-\frac{length\;of\;treated\;seedlings}{length\;of\;control\;seedlings}\times 100$$

### 4.3. Isolation and Purification of the Growth-Inhibitory Substances

*Polygonum chinense* plant powder (2.1 kg) was extracted as described in Section 4.1 using 10 L of the exact ratio of distilled water and MeOH (ratio 30:70, *v*/*v*) and 10 L of MeOH. A rotary evaporator was then used to condense the extract at 40 °C to produce aqueous residues. A 1 M phosphate buffer solution was then used to bring the concentrated residues to pH 7.0. The equal volume of ethyl acetate was partitioned five times (150 mL each time) to obtain an aqueous (distilled water) fraction and an ethyl acetate fraction. The effects of the distilled water fraction and the ethyl acetate (EtOAc) fraction on growth suppression were evaluated using a cress assay. After being treated overnight with anhydrous sodium sulfate (Na_2_SO_4_ used to remove water), the ethyl acetate fraction was filtrated and subjected to the next purification procedures, and this fraction was then evaporated until completely dry.

The EtOAc fraction continued to be separated by a silica gel column (60 g of silica gel, spherical, 70–230 mesh: Nacalai Tesque, Kyoto, Japan.) eluted stepwise with the ratio of ethyl acetate in *n*-hexane 20:80 to 80:20 (*v*/*v*, 150 mL per step), 150 mL of ethyl acetate, and 300 mL of methanol. A cress assay was used to assess the biological effect of these nine fractions. The biological activity obtained from the separation of the silica gel column showed that there were two active fractions eluted with 30:70 and 20:80 EtOAc in *n*-hexane (*v*/*v*). The fraction eluted by 70% EtOAc in *n*-hexane was first conducted to isolate the active substances. The residues were evaporated to dry and then separated using a column of Sephadex LH-20 (80 g; Sigma-Aldrich, Burlington, VT, USA). Five different concentrations of aqueous methanol (20, 40, 60, 80%, *v*/*v*, 150 mL each step, and 300 mL of methanol), were loaded onto the Sephadex column. These fractions were evaporated until dry, and a growth assay of cress was carried out to determine how these fractions affected the biological processes. An inhibitory active fraction was eluted by 40% of aqueous methanol (F2). The active fraction was again evaporated until dry and diluted with 20% (*v*/*v*) aqueous methanol and loaded onto a reverse-phase of C_18_ Sep-Pak cartridge (YMC Co., Ltd., Kyoto, Japan). Seven different concentrations of aqueous methanol (20 to 70%, *v*/*v*, 15 mL each step, and 30 mL of methanol) were loaded on the C_18_ Sep-Pak cartridge. The most active fraction was eluted in fraction 3 (40% aqueous methanol).

Fraction 3 was then isolated by running reverse-phase of high-performance liquid chromatography (HPLC; I.D. ODS, 500 × 10 mm, Shimadzu Corporation, Kyoto, Japan) eluted with 40% (*v*/*v*) aqueous methanol at a flow rate of 1.5 mL/min. The active peak was detected at a wavelength of 220 nm in a 40 °C oven at a retention time of 95–99 min. The active peak was purified once again by running HPLC (S-5 µm, 4.6 mm × 250 mm I.D., Inertsil^®^ ODS-3; GL Science Inc., Tokyo, Japan) eluted with 40% (*v*/*v*) aqueous methanol at a flow rate of 0.8 mL/min. Compound **1** was detected in a retention time of 16–17 min and an oven temperature of 40 °C at 220 nm.

Another compound was isolated from the silica gel column (fraction 7), the Sephadex LH-20 column (fraction 2), the C_18_ Sep-Pak cartridge column (fraction 2), and running HPLC (500 × 10 mm I.D. ODS AQ-325; Shimadzu Corporation, Kyoto, Japan) eluted 50% aqueous methanol at a flow rate of 1.5 mL/min, and detected in a retention time of 84–89 min, at an oven temperature of 40 °C at 220 nm. Compound **2** was then purified once again at a flow rate of 0.8 mL/min with 35% aqueous methanol by running reverse-phase HPLC (S-5 µm, 4.6 × 250 mm I.D., Inertsil ^®^ ODS-3; GL Science Inc.), and the pure peak was obtained at the retention time of 38–41 min. Finally, spectral analysis was used to characterize the chemical structures of these two compounds. A schematic diagram of the isolation procedure of the two substances is presented in Figure 3.

### 4.4. Bioassay of the Identified Compounds

The two compounds were dissolved in 3 mL of methanol separately to obtain solutions at five concentrations of 0.03, 0.1, 0.3, 1, 3, and 10 mM, which were prepared and added to Petri dishes (2.8 cm diameter) lined with filter paper. Cress seeds were used to test the inhibitory effect of compound 1, and sprouted seeds of Italian ryegrass (monocot) and seeds of alfalfa and cress (dicots) were used to test the inhibitory effect of compound **2** as described in Section 4.2. Each treatment was conducted with three replicates (*n* = 30).

### 4.5. Spectral Data

A JASCO P-1010 polarimeter was used to measure the optical rotation. All NMR spectroscopic data were recorded on a Bruker AVANCE III 500 MHz NMR spectrometer. Chemical shifts were reported relative to the residual solvent signal (CD_3_OD: δ_H_ 3.31). HRESIMS was performed using a Thermo Scientific Orbitrap Exploris 240 mass spectrometer.

### 4.6. Statistical Analysis

The assay experiments were arranged in a completely randomized block design (10 seeds for each treatment) with three replicates. The results are shown as mean ± SE (standard error). The ANOVA and Tukey’s honestly significant difference (HSD) test for multiple comparisons were performed using Version 16.0 of the Statistical Package for the Social Sciences, SPSS, IBM, Armonk, NY, USA, with a significance level of 0.05. The I_50_ values were analyzed using GraphPad Prism Version 6.0 software package, San Diego, CA, USA.

## 5. Conclusions

The aqueous methanol extracts of the *Polygonum chinense* plant showed allelopathic activity. The two active allelopathic substances were isolated from the silica gel column of two different fractions and characterized as dehydrovomifoliol and loliolide. These two compounds showed allelopathic effects against each test plant. The findings of our study showed that these two compounds possess allelopathic potential and may contribute through the decomposition of plant residues and possibly act as allelopathic agents. However, more research is needed to examine the mechanisms underlying the allelopathy of *P. chinense*, the role of allelochemicals of different functional groups, and the long-term effects of allelopathy in soil residues.

## Figures and Tables

**Table 1:** Concentrations necessary for the *P. chinense* extracts to inhibit the seedling growth of the test plants by 50% (I_50_ values).

Test Plant	I_50_ Value (g DW Equivalent Extract/mL)	
	Shoot	Root
Alfalfa	0.0035	0.0049
Italian ryegrass	0.0043	0.0052

**Table 2:** Concentrations required for dehydrovomifoliol to inhibit the growth of the cress seedling shoots and roots by 50% (I_50_ values).

Test Plant	I_50_ Value (mM)	
	Shoot	Root
Cress	2	1.2

**Table 3:** Concentrations required for loliolide to inhibit the growth of the test plant seedlings by 50% (I_50_ values).

Test Plant	I_50_ Value (mM)	
	Shoot	Root
Cress	0.15	0.33
Alfalfa	2.33	2.23
Italian ryegrass	0.80	1.90

## Data Availability

Not applicable.
